# Asthma heterogeneity and severity

**DOI:** 10.1186/s40413-016-0131-2

**Published:** 2016-11-29

**Authors:** Tara F. Carr, Eugene Bleecker

**Affiliations:** 1 Asthma and Airway Disease Research Center, University of Arizona, 1501 N Campbell Ave, Tucson, AZ 85724-5030 USA; 2 Center for Genomics and Personalized Medicine Research, Wake Forest School of Medicine, Winston-Salem, NC USA

**Keywords:** Severe asthma, Phenotype, Heterogeneity

## Abstract

Asthma is a common, chronic inflammatory airways disease characterized by a clinical syndrome of bronchial hyperresponsiveness, inflammation, and reversible airflow obstruction. Individuals with asthma can vary widely in clinical presentation, severity, and pathobiology. The incident factors, pathogenesis, prognosis, and treatment of asthma remain incompletely understood. Utilizing measurable characteristics of asthmatic patients, including demographic, physiologic, and biologic markers, can however identify meaningful phenotypic categories in asthma. Identification of these phenotypes may help improve precision therapeutics targeted toward an individual’s’ disease, and may identify strategies for preventing progression of disease severity.

## Background

Asthma is a chronic inflammatory disease of the airways. Individuals with asthma may experience recurrent wheezing, dyspnea, chest tightness, and cough. These symptoms reflect episodes of reversible airflow obstruction, which may remit spontaneously or with treatment. Over time, many asthmatics experience progressive airway remodeling, leading to an incompletely reversible, or fixed, airflow obstruction. Further, inflammation in the asthmatic airway induces airway bronchial hyper-responsiveness to a variety of allergic, infectious, or irritant stimuli.

### Public health impact of asthma

Asthma is a very common chronic disorder. Asthma severity can range from intermittent to severe; more severe asthma is associated with significant morbidity and mortality. Further, asthma prevalence is increasing with time [1], perhaps due to better recognition and phenotyping. It is estimated that, in the United States in 2013, asthma affected 16.5 million adults and 6.1 million children, reflecting 8.3% and 7.0% of the population, respectively [2]. Approximately half of those individuals experienced an asthma attack, which is defined as sudden worsening of asthma symptoms due to bronchoconstriction, and when severe, hyperinflation and “air trapping” [3]. Asthma is the leading cause of absenteeism in children in the United States, causing approximately 50% of children to miss at least one school day each year, and one in three adults to miss at least 1 day of work. Three out of five asthmatics are forced to limit their usual activities because of this disease.

Asthma remains a prevalent disease worldwide. Estimates from worldwide analyses such as the Global Burden of Disease Study from the Forum of International Respiratory Societies suggest that asthma affects at least 235–334 million individuals [4, 5]. Using data from the International Study of Asthma and Allergies in Childhood surveys, approximately 14% of the world’s children suffer from asthma in any given year. Latin American and English-speaking countries of Australiasia, Europe, North America, and South America have the highest prevalence of childhood asthma, estimated at over 20% [6]. Reported asthma symptoms in children increased from 1993 to 2003 in low- and middle-income countries. Estimates of asthma prevalence in adults are more difficult to obtain. Approximately 8.6% of adults worldwide between the ages of 18–45 have asthma symptoms. The morbidity and mortality burden of disease, however, disproportionately affects older adults [5].

Global measures of disability rank asthma 14^th^ in number of years lost to asthma-associated morbidity and mortality [7]. This most significantly affects individuals in some countries of Europe, Central and South America, Africa, and Austrailasia. Annually in the United States, asthma accounts for approximately 15.5 million outpatient health care visits, 1.8 million emergency department visits, and 439,000 hospitalizations, costing the US $56 billion each year, or roughly $3259 per person [8]. In a European study from 2011, the estimated total cost of asthma in adolescents and adults was €19.3 billion [9]. In the Asia-Pacific region, the estimated direct and indirect cost of asthma per person range from $184 to 1189. In the United States in 2013, 3630 individuals died from asthma, or nine people per day [1, 8]. These data suggest asthma is often poorly controlled, despite the availability of pharmacologic therapies that are recommended in National and International Asthma Guidelines [10–12].

### Development of asthma

**Fig. 1:**
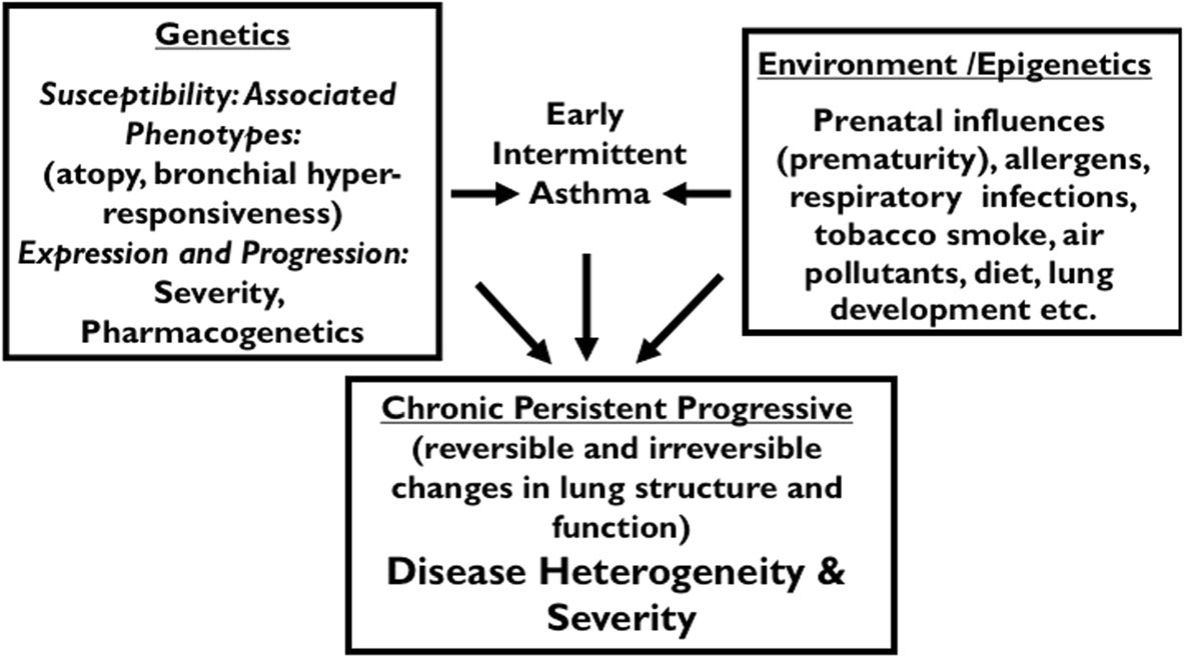
Gene-environment interactions in susceptibility and severity of asthma

An individual’s susceptibility to the development of asthma, or to severity of asthma, are likely determined by an interaction of host or genetic characteristics that interact with environmental exposures. For example, specific genotypes can confer susceptibility to developing wheezing with rhinovirus exposure [13], atopy, or responsiveness to bronchodilator therapy [14, 15]. Currently, there are a number of genes that are associated with asthma susceptibility [16]. An important question in whether these or different genes influence asthma progression and severity. Environmental exposures, including prenatal influences [17], allergens [18, 19], respiratory infections [20–22], cigarette smoke [23], and air pollution [24] are implicated in the development of asthma. Cumulative environmental exposures may lead to persistent, progressive disease with potentially irreversible changes in lung structure and function. These concepts are illustrated in Fig. 1 which describes the interaction between genetics and environment in the development and progression of asthma. Because of differences in the influence of genes and environment, there is a wide range of disease heterogeneity and severity in asthma.

### Assessment of asthma

All that wheezes is not asthma, and all asthma does not wheeze. Accurate diagnosis of asthma is important, as treatment will benefit both morbidity and mortality from this disorder. As many non-asthmatic diseases have overlapping clinical findings with asthma, accuracy of clinical diagnosis is critical for planning appropriate treatment strategies. The Global Strategy for Asthma Management and Prevention 2015 report update [12] and the National Institutes of Health Guidelines for the Diagnosis and Management of Asthma Expert Panel Report-3 [10] provide recommendations for the diagnosis of asthma. In addition to obtaining a detailed history of symptoms and physical exam, these guidelines suggest obtaining studies of lung function such as spirometry to measure forced expiratory volume in 1 s (FEV1) and forced vital capacity (FVC) measurement, as the FEV1/FVC ratio objectively measures airflow obstruction. Additional pulmonary function testing such as diffusing capacity, lung volumes, or bronchoprovocation studies to support or refute the asthma diagnosis. Comorbidities and alternate diagnoses should be evaluated when symptoms are atypical or not responding to therapy.

### Treatment of non-severe asthma

Goals of asthma treatment are multifaceted. A combination of controller and rescue therapy for asthma usually allows an individual to achieve and maintain control of asthma symptoms. Control of asthma should confer a normal day-to-day activity level, including exercise capacity. Treatment of asthma may prevent the development of irreversible airflow limitation and allow maintenance of best possible pulmonary function. Adequate control of asthma, by definition, should prevent exacerbations and limit mortality due to asthma [10, 12]. Importantly, treatment should also identify and minimize medication side effects.

The Global Strategy for Asthma Management and Prevention 2015 report update [12] and the National Institutes of Health Guidelines for the Diagnosis and Management of Asthma Expert Panel Report-3 [10] also provide a framework for the treatment of asthma. These guidelines emphasize evaluation of impairment and risk, with ongoing assessment of control. The domains of impairment and control focus on assessment of symptom frequency, frequency of use of rescue medications, impact on activity levels, and lung function. The risk domain identifies risk of exacerbations and adverse outcomes utilizing an individuals’ history of exacerbations and lung function, with a goal of prevention of future exacerbations or fixed airflow limitation. The severity of asthma as measured through these domains is then used to guide treatment.

A stepwise approach to therapy is recommended, which highlights use of controller medications, particularly inhaled corticosteroids, then titrating doses or adding additional therapies as needed to achieve the necessary level of symptom control. At every level, assessment of proper inhaler device techniques, adherence to therapy, environmental control, and use of rescue inhalers for quick relief of sudden symptoms are recommended. Well recognized, however, is the inter-individual variability in response to each treatment [25, 26], reflecting the heterogeneity of disease which exists across severity groups.

### Severe asthma

#### Task force definitions of severe asthma

The American Thoracic Society and European Respiratory Society released a Task Force document in 2014 entitled “International ERS/ATS Guidelines on Definition, Evaluation and Treatment of Severe Asthma [11].” The purposes of this document include defining severe asthma and treatment-resistant asthma; discussing phenotypes of severe asthma with respect to genetics, natural history, pathobiology, and physiology; outlining evaluation of a patient with severe asthma; and providing recommendations for treatment of severe asthma in children and adults. Assuming asthma diagnosis is accurate and comorbidities are being addressed, severe asthma is defined as asthma that requires treatment with guidelines-suggested medications such as high dose inhaled corticosteroids and a second controller for the previous year, and/or systemic corticosteroids for at least half of the previous year, to prevent it from becoming ‘uncontrolled’ or which remains ‘uncontrolled’ despite this therapy. Uncontrolled asthma is defined as the presence at least one of the following characteristics: persistently poor symptom control, two or more exacerbations requiring bursts of systemic corticosteroids in the preceding year, at least one serious exacerbation requiring hospitalization in the previous year, or chronic airflow limitation of FEV1 < 80% predicted with FEV1/FVC ratio less than the lower limit of normal [11].

#### Evaluation of patients with severe asthma

Individuals with severe asthma should undergo a careful systematic assessment to confirm this diagnosis. Lung function testing is utilized to confirm airflow obstruction and to measure reversibility or variability of airflow obstruction. Bronchoprovocation testing, such as with methacholine inhalation or exercise, may also be utilized. Medication noncompliance or poor inhaler technique can be identified in many severe asthmatics [27, 28]. Atopy and unregulated allergic exposures, such as ongoing house dust mite or cockroach exposure in an individual with sensitization to these antigens, may contribute to severe asthma, particularly in children [29]. Chronic rhinosinusitis is a very common comorbidity of asthma and contributes to disease severity [30, 31]. Obesity, obstructive sleep apnea, and psychological factors may contribute to asthma severity or perception [11, 32]. Symptomatic gastro-esophageal reflux disease is common in asthmatics, but the effect of treatment on asthma control or severity is currently unclear. The contribution of tobacco smoke exposure, hormones, and medication use should be carefully considered, as avoidance of the offending agent can confer major benefits on asthma control [11].

#### Treatment of patients with severe asthma

Inhaled corticosteroids remain the mainstay of asthma treatment, particularly in mild to moderate disease. By definition, those individuals with severe asthma require high doses of corticosteroid to control disease, and often remain symptomatic despite this therapy. Further, a subset of severe asthmatics is relatively corticosteroid insensitive, with relative or complete lack of clinical improvement from treatment with inhaled or systemic corticosteroids. While corticosteroid insensitivity seems more common in those with vitamin D deficiency or obesity, eosinophilic or type-2 inflammation-high asthma may have a relative benefit from steroids when compared to those with non-eosinophilic, non-type-2 inflammation [33].

**Fig. 2:**
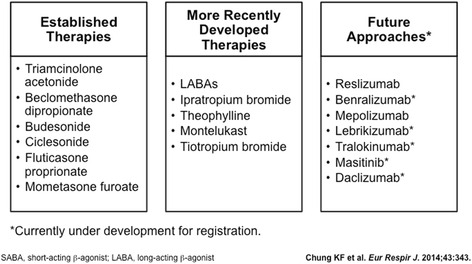
European respiratory society and American thoracic society task force; *severe or therapy-resistant asthma: therapy*

Other controller therapies may benefit some individuals with severe asthma. Beta-agonists provide smooth muscle relaxation and bronchodilation through beta-adrenergic receptors. While short acting and long acting beta-agonists are used in asthma, concern that these drugs may contribute to asthma treatment failure, particularly in individuals with genetic differences in the beta-adrenergic receptor, may impact use [15]. However, recent results of United States Food and Drug Administration-mandated safety studies with inhaled corticosteroid-long acting beta agonist combination therapy do not show evidence of adverse effects [34, 35]. Leukotriene modifiers may benefit severe asthmatics with aspirin exacerbated respiratory disease. Anticholinergics block smooth muscle contraction through inhibition of the muscarinic receptor-3. The long-acting muscarinic antagonist Tiotriopum bromide has shown benefit some individuals with severe asthma [36, 37]. These treatments, as well as potential future approaches, are highlighted in Fig. 2.

Biological therapeutics, those with a specific pathobiological target, have been and continue to be developed for use in severe asthma with particular phenotypes. Three are available currently in the United States for clinical use. Omalizumab, a monoclonal anti-Immunoglobulin E antibody, may be beneficial for some allergic asthmatics uncontrolled on therapy [38]. Mepolizumab and Reslizumab, both monoclonal anti-IL5 antibodies, reduce asthma exacerbations in those with severe eosinophilic asthma [39, 40], Different treatments, particularly for those with both-type 2 and non-Th2 inflammatory asthma, are under active development [41–43].

### Asthma heterogeneity

With a developing understanding of the marked heterogeneity within the disease of asthma, we hypothesize, and expect to confirm, that the heterogeneity of asthma is attributable largely to individuals’ genetic and epigenetic variability, mediated by certain environmental exposures. Environmental exposures are highly dependent on regional characteristics with varying climatic conditions, geography and population distributions. This variability in turn drives the immunologic mechanisms, or endotype, that confer the pathobiological and physiologic characteristics of asthma, the phenotype, as measured in the clinical setting. Importantly, our understanding of this variability and the mechanisms causing this disease may facilitate the development of interventions for primary prevention, disease modification, and precision therapeutics.

Hypothesis-driven univariate approaches to phenotyping have been utilized to clarify differences among groups of asthmatics. This type of approach defines groups based on the presence or quality of one variable, which is chosen to support testing a specific hypothesis. Disease severity may be the most straightforward, if not oversimplified, way of delineating disease phenotype. As anticipated, and likely as a result of the definitions of severe asthma, groups with severe asthma can be distinguished from non-severe asthmatics in terms of disease duration, symptomatology, health care utilization, lung function, and comorbidities [44–47]. However, it is well recognized that disease heterogeneity is present and vitally important among these severity classes, particularly among the more severe asthmatics [48] wherein cellular characteristics and airway remodeling have been long shown to confer different physiologic subtypes. Phenotypic characterization solely by disease severity therefore lacks the granularity to understand and delineate subtypes of asthma.

Other clinical characteristics have been assessed using hypothesis-driven univariate approaches. Reduction in mid forced expiratory flow rates (FEF25-75), as well as in FEV1, have been shown to be independently associated with markers of asthma severity, including ICU admissions, persistent or nocturnal symptoms, peripheral blood eosinophilia, and bronchial hyperreactivity [49]. A striking relationship between age and the probability of severe asthma was identified, particularly in men, increasing with duration of disease and from ages 18 to 45 [47, 50]. Airway mast cell phenotype and activation may contribute to phenotype and clinical characteristics. Indeed, mast cells containing both tryptase and chymase have been identified as the predominant phenotype in patients with severe asthma, whereas mast cells containing only tryptase are identified in biopsies from individuals with mild disease [51].

Inflammatory mediators within the airway may also be used for disease phenotyping. These inflammatory markers, present in sputum supernatant or bronchoalveolar lavage, may be related to cellular patterns that then relate to clinical phenotypes [52], or to disease characteristics such as eosinophilia, neutrophilia, airway bronchial hyperresponsiveness, and bronchodilator response [53]. Interestingly, when examining broncoalveolar lavage of children with asthma, while markers such as IL-13 and IL-6 can differentiate asthmatics from controls, and other cytokines can distinguish moderate from severe asthma, severe asthma itself does not have a clearly TH1 or TH2 inflammatory pattern [54]. This further underscores the heterogeneity of severe asthma.

Finally, technology to measure gene expression such as microarray and RNA-seq can provide insight into abnormally expressed pathways. Bronchial airway epithelial gene expression patterns were assessed in relationship to the clinical biomarker fractional exhaled nitric oxide (FeNO). Using a subset of genes that correlated with FeNO, subject clusters can be identified as having distinct clinical and molecular characteristics [55].

### Model-free multivariate (Unbiased Cluster) approaches

Unbiased approaches to phenotyping utilize computer algorithms to evaluate hypothesis-free relationships among many clinical and biological characteristics. The resultant clusters, because they were created in an unbiased manner, can provide novel insights into asthma phenotypes.

**Fig. 3:**
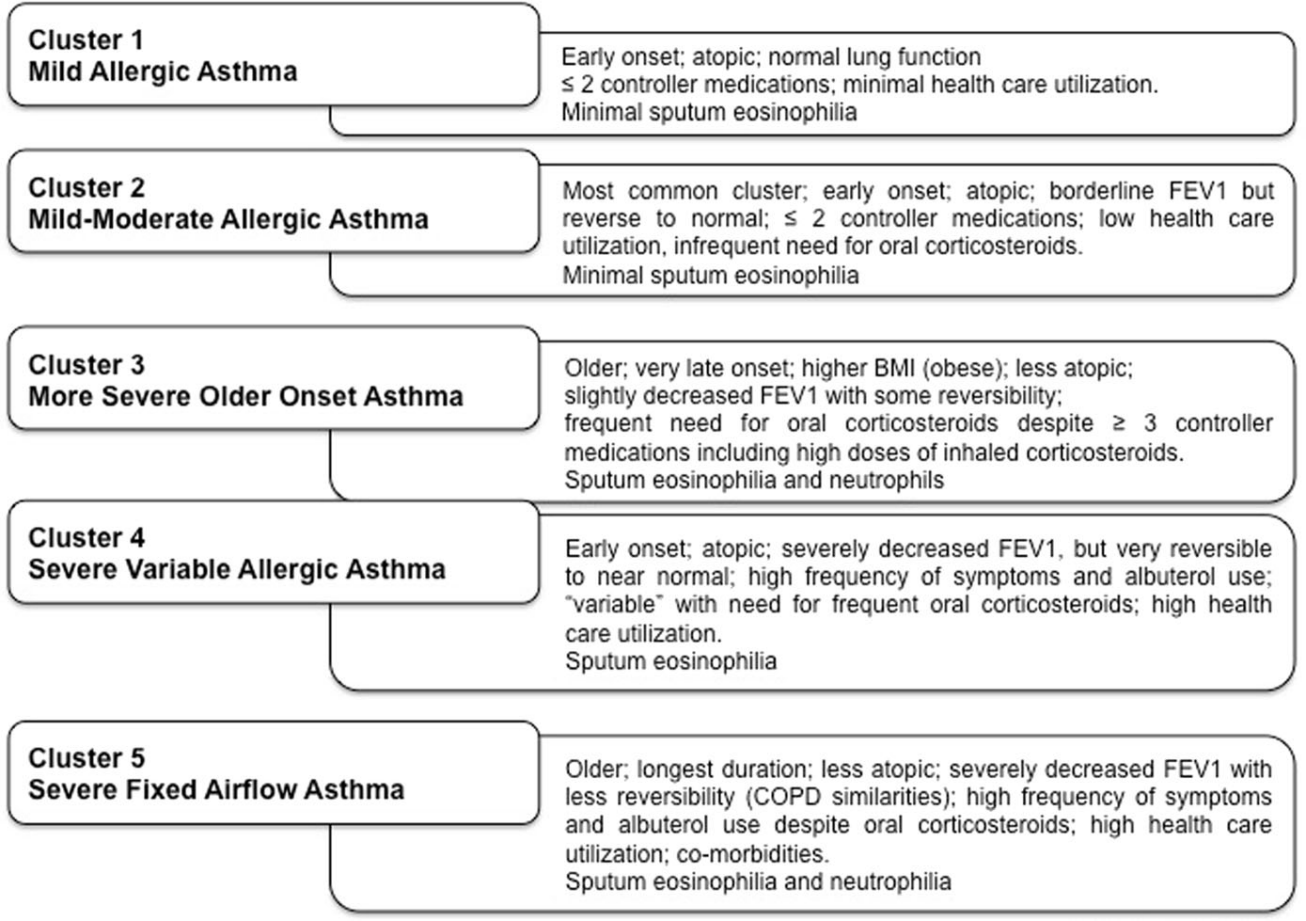
Severe asthma research program clinical clusters

The National Institutes of Health-sponsored Severe Asthma Research Program (SARP) enrolled and carefully assessed large cross-sectional cohorts of mild, moderate, and severe asthmatic adults and children. Unsupervised hierarchical cluster analysis performed on clinical and physiologic data from ~700 adult asthmatics in the SARP cohort identified five clusters of asthmatic subjects (Fig. 3) [47, 56]. Clinical clusters 1, 2, and 4 contain early onset, atopic asthmatics of increasing disease severity and worsening lung function. Cluster 3 is characterized by older, obese women with late-onset non-atopic asthma, with moderate lung function deficits and frequent exacerbations. Cluster 5 is characterized by later onset non-atopic asthma with more severe, irreversible airflow obstruction and high health care utilization. The most influential variables in forming these clusters include gender, age of asthma onset, asthma duration, use of inhaled beta-agonists and corticosteroids, and lung function pre- and post-bronchodilator administration [57].

With an unrelated cohort, investigators from Leicester likewise examined adult asthmatics through cluster analysis, revealing similar phenotypes of benign (mild) asthma, early onset atopic asthma, early onset symptom predominant asthma, obese non-eosinophilic asthma, and late onset inflammation predominant asthma [58]. The reproducible findings of these and other unrelated cohorts support these phenotypes as relevant [44, 59, 60].

Unsupervised cluster analyses were also performed on 161 subjects in the pediatric asthmatic cohort from SARP [61]. Four clusters were identified. Cluster 1 consists mainly of mild, later onset, less atopic asthma with normal lung function. Clusters 2, and 3 represent the spectrum of early onset, atopic asthma with increasing severity and worsening lung function. Cluster 4 identified a subset with more severe, fixed airflow obstruction and the highest health care utilization. These clusters have similarities to those seen in the adult SARP analyses.

Unsupervised cluster analysis was similarly utilized by researchers from the Trousseau Asthma Program in Paris, France [62] to identify phenotypic clusters in a pediatric severe asthma cohort of 315 subjects. Clinical and inflammatory markers were included in these analyses. Three clusters were identified: one of mild asthma, one of highly atopic asthma with eosinophilia and severe exacerbations, and one of higher body mass index, neutrophilia and more severe airflow obstruction. Despite the differences between the SARP and Trousseau cohorts, the clusters have features that generally overlap: SARP cluster 1 similar to the “mild” cluster, SARP cluster 3 to the “atopic severe” cluster and SARP cluster 4 to the “airflow obstruction” cluster.

Sputum cellular characteristics can identify patterns of airway inflammation and may have clinical utility. For example, individuals with sputum eosinophilia are likely to derive benefit from use of inhaled corticosteroids [63]. Phenotyping by cellular characteristics also can identify groups with differences in clinical and inflammatory markers. Airway neutrophilia has been associated with severe asthma defined by low lung function and use of high dose inhaled or oral corticosteroids [64]. Similarly in the SARP cohort, when using pre-defined normal and elevated cell counts, in the absence of cluster analysis, cellular asthmatics with elevated sputum eosinophilia (≥2%) and neutrophilia (≥ 40%) tended to have lower lung function, increased symptoms and health care utilization when compared with others [52].

A further examination of the adult SARP data integrated inflammatory cellular measures with the clinical variables in an unsupervised cluster analysis. Four phenotypic clusters were identified, which represented a severity spectrum from those with mild-to-moderate allergic disease (SARP clusters 1,2), having predominantly paucigranulocytic or eosinophilic sputum, to those with moderate-to-severe asthma or impaired lung function, most of whom had significant sputum neutrophilia with or without significant eosinophilia (SARP clinical clusters 3, 4, and 5) [65]. Importantly, the more inflammatory and severe clusters had markedly increased asthma medication use and health care utilization, including bursts of systemic corticosteroids and hospitalizations [57, 65].

Data collected from longitudinal cohorts can also be used for unsupervised cluster analyses, leveraging the power of the longitudinal design to provide insight into the variable patterns of disease over time. Analyses of pediatric birth cohorts have identified clusters of wheeze, atopy, or other characteristics that are associated with risk for asthma-related outcomes into the teenaged years. For example, the Avon Longitudinal Study of Parents And Children (ALSPAC) study collected data on wheezing at multiple time points from birth to age 7 years, for 6265 children in the United Kingdom [66]. The authors utilized wheeze data in latent class analysis to describe patterns of early wheeze, then examined clinical characteristics of individuals in these classes. Associations with atopy, airway hyper-responsiveness, and lung function abnormalities were seen in intermediate and late onset wheezing. These findings were similar to those from analyses of the Dutch Prevention and Incidence of Asthma and Mite Allergy (PIAMA) study, a multicenter birth cohort that enrolled 4146 pregnant women [67]. A latent class analysis of the PIAMA data identified 5 phenotypes of childhood wheeze, similar to those seen in ALSPAC [68]. The ALSPAC cohort was again assessed after age 16; latent class analysis identified early onset persistent wheeze to confer risk of lung function abnormalities.

The Manchester Asthma and Allergy Study is an unselected birth cohort of over 1000 children with periodic lung function and assessments of atopy and other clinical characteristics. Principal component analysis was performed using twenty one variables available at up to 5 years of age; patterns of wheeze and cough components were significant contributing components to the groups [69]. With the availability of 8-year old data for this cohort, a latent class analysis was performed, which identified differences in lung function trajectories over time among the classes, as well as more severe asthmatics with exacerbation risk in the persistent troublesome wheezing group [70].

In a population-based longitudinal cohort that enrolled 1,650 preschool children in Leicestershire, United Kingdom, early life wheeze and atopy data were used for latent class analysis [71]. The three wheeze and two cough phenotypes identified from early life data were assessed for associations with school age respiratory outcomes. The atopic persistent wheezers from early life had highest rates of current or frequent wheeze at ages 8-13. These authors identified a validation cohort of 6970 children born in a different county of the United Kingdom, for whom atopy and respiratory assessments were available at ages 8–13 in approximately 900. Latent class analyses revealed five groups with very similar characteristics to the groups seen in the original cohort [72].

Unbiased analyses from longitudinal cohorts indeed complement those of the cross-sectional cohorts. Despite slight differences among the clusters in each cohort, these unsupervised analyses ultimately identify clearly that asthma phenotypes vary by atopy, age of wheeze onset, clinical and physiologic characteristics. The stability of these clusters into adulthood is not well known, however, and the potential for progression from milder asthma to more severe disease, or vice versa, needs further elucidation.

## Conclusion

We can easily recognize the clinical syndrome of asthma, presenting as symptoms of reversible airflow obstruction with airway hyper-reactivity and inflammation. More severe asthma is associated with exacerbations that cause a significant degree of morbidity and even mortality. However, the incident factors, pathogenesis, prognosis, and treatment of asthma remain incompletely understood. Utilizing measurable characteristics of asthmatic patients, including demographic, physiologic, and biologic markers, can identify meaningful phenotypic categories of asthma. These phenotypes, while providing a helpful albeit partial understanding of disease state, can be further leveraged toward endotypic characterization, with the ultimate goals of identifying preventative strategies and improving precision therapeutics targeted toward an individual’s disease.

## Abbreviations

ALSPAC: Avon longitudinal study of parents and children
FeNO: Fractional exhaled nitric oxide
FEV1: Forced expiratory volume in 1 s
FVC: Forced vital capacity
PIAMA: Prevention and incidence of asthma and mite allergy
SARP: Severe asthma research program
